# Experimental Analysis of Hot-Mix Asphalt (HMA) Mixtures with Reclaimed Asphalt Pavement (RAP) in Railway Sub-Ballast

**DOI:** 10.3390/ma16041335

**Published:** 2023-02-04

**Authors:** Nicola Fiore, Salvatore Bruno, Giulia Del Serrone, Franco Iacobini, Gabriella Giorgi, Alessandro Rinaldi, Laura Moretti, Gian Marco Duranti, Paolo Peluso, Lorenzo Vita, Antonio D’Andrea

**Affiliations:** 1 Department of Civil, Constructional and Environmental Engineering, Sapienza University of Rome, Via Eudossiana 18, 00184 Rome, Italy; 2 Technical Department, Rete Ferroviaria Italiana S.p.a., Piazza della Croce Rossa 1, 00161 Rome, Italy

**Keywords:** reclaimed asphalt pavement (RAP), life cycle impact analysis (LCIA), asphalt recycling, sustainability, rejuvenator

## Abstract

Environmental safeguards promote innovative construction technologies for sustainable pavements. On these premises, this study investigated four hot mix asphalt (HMA) mixtures—i.e., A, B, C, and D—for the railway sub-ballast layer with 0%, 10%, 20%, and 30% reclaimed asphalt pavement (RAP) by total aggregate mass and a rejuvenator additive, varying the bitumen content between 3.5% and 5.0%. Both Marshall and gyratory compactor design methods have been performed, matching the stability, indirect tensile strength, and volumetric properties of each mixture. Dynamic stiffness and fatigue resistance tests provided mechanical performances. Laboratory results highlighted that the RAP and the rejuvenator additive increase the mechanical properties of the mixtures. In addition, the comparative analysis of production costs revealed up to 20% savings as the RAP content increased, and the life cycle impact analysis (LCIA) proved a reduction of the environmental impacts (up to 2% for resource use-fossils, up to 7% for climate change, and up to 13% for water use). The experimental results confirm that HMA containing RAP has mechanical performances higher than the reference mixture with only virgin raw materials. These findings could contribute to waste management and reduce the environmental and economic costs, since the use of RAP in the sub-ballast is not, so far, provided in the Italian specifications for railway construction.

## 1. Introduction

In the last decades, the increase in railway traffic loads and repetitions forced the development of innovative and high-performance technologies [1]. During its service life, the permanent way should maintain track geometry, distribute the loads on the sleepers, and require minimal, safe, and fast maintenance works [2,3,4]. Such requirements are even more stringent for high-speed track structures [5]. In the early 2000s, the Italian railway company (i.e., Rete Ferroviaria Italiana) first introduced an asphalt concrete sub-ballast layer (sub-ballast) under the ballasted track [6]. Sub-ballast is an alternative solution to the traditional blanket to distribute the load on the lower layers, prevent contamination between ballast and subgrade, and reduce the stress on the subgrade [7]. A bound layer can prevent mud pumping and protect the railway subgrade from erosion and climatic changes to slow down the decay process [8], especially in cold regions [9]. Moreover, sub-ballast can limit vertical rail deformations and dampen vibrations [10,11,12,13,14]. In the literature, several analytical and numerical models to investigate railway track performance [15] demonstrated the effectiveness of asphalt layers to increase stiffness and bearing capacity [16] of the substructure [17], while the dynamic effects still should be deepened [18]. Fang et al. [19] identified four asphalt trackbeds (i.e., asphalt underlayment, asphalt overlayment, surface asphalt mixture impermeable, and asphalt stabilized ballast) that differ for functional and structural properties of the bitumen-bound materials. Global experiences demonstrated that the construction of sub-ballast [20,21] is a strategic solution for high-speed and high-capacity lines [11,22,23], although it has higher environmental and economic costs [24,25]. In the last few years, extensive efforts to address environmental protection led to the adoption of low-impact mixing and laying technologies and to the use of secondary and/or high-performance raw materials, low-temperature asphalt mixtures, modified bitumen, and high-performance additives [26,27,28,29,30]. In particular, mixtures modified with rubber and polymer returned interesting results in terms of fatigue resistance [31,32], mechanical properties [33,34], and damping vibrations [35]. Moreover, geofiber reinforced soil layers have been investigated [36] and were found to obtain a cost-effective alternative to soil improvement.

The use of reclaimed asphalt pavement (RAP) as recycled aggregate and rejuvenated binder has become popular worldwide, and RAP is one of the most reused building materials [25,37]. Its blending efficiency depends on several variables such as the effective binder content, the quality of the aged bitumen, and the production process [38]. In the literature, several studies investigated the feasibility of using RAP to produce recycled HMA [39] and lots of mixtures with different RAP contents have been investigated to identify the optimum bitumen content (OBC) [40,41].

This technology implies significant environmental gains [42,43]:

- Reduced depletion of both natural fossils and elements resources;
- Reduced landfill disposal for construction and demolition waste;
- Reduced impacts for transporting asphalt to roadworks;
- Reduced costs to buy and transport natural aggregates and bitumen.

On the other hand, the contaminant leaching from recycled asphalt pavements [44] and the production thermal processes [45] have been investigated to identify critical issues of recycling technology in asphalt plants. In particular, the production of recycled mixtures requires the addition of a rejuvenating agent or rejuvenator to restore the RAP binder properties [46] and reduce the content of virgin bitumen [47]. RAP is not preheated or heated to a lower temperature than natural aggregates because the preheating process causes overaging of the binder included in RAP [48,49].

However, bitumen oxidation due to different aging conditions (e.g., mixing, laying, and environmental conditions) is still an open challenge in the use of RAP to design new mixtures [50]. Chemical and rheological properties of rejuvenated binders can differ from those of virgin bitumen and require further analyses [51]. Nevertheless, recent studies demonstrated that it is possible to produce mixtures with a high percentage of RAP [52]. Shu et al. [53,54] observed the benefits of RAP to improve mixture moisture resistance. Furthermore, Zhu et al. [55] tested 40% recycled mixtures with an increase in moisture resistance and dynamic modulus. Valdès et al. [56] investigated how bitumen and RAP affect stiffness modulus, indirect tensile strength (ITS), cracking behavior, and fatigue resistance of hot mixtures. Bernier et al. [57] tested the rutting susceptibility. Kucera et al. [37] surveyed trial sections with the asphalt trackbed layer with up to 70% of RAP. The structures provided resistance against lateral, longitudinal, and vertical movement of ties and rails, and protected the subgrade from temperature variation and rainwater run-off.

The use of RAP is a useful strategy for increasing the environmental sustainability of construction materials because it reduces the amount of virgin bitumen, the exploitation of quarries, and the disposal of waste from road pavement rehabilitation. Although the results in the literature highlight the suitability for hot mix asphalt with RAP, deep investigations are required to establish threshold limits of usable RAP [37] without decay of performances. In this study, the physical characteristics and compositions of both aggregates and milled material were studied to identify their mechanical, volumetric, and resistance properties. The mixtures composed of aggregates and RAP and additive were designed by varying the relative percentages of these components to optimize the sub-ballast mix design. The Marshall mix design and the volumetric mix design using a gyratory press allow the identification of the OBC. Then, we investigated the use of RAP in remarkable quantities within an HMA to be used as a railway sub-ballast. The identification of the higher content of RAP was performed by comparing mechanical, volumetric, and strength properties of the tested mixtures through tensile strength, fatigue resistance, and stiffness modulus. At the same time, an additional economic and environmental impact analysis provided the best solution. Finally, the results proved the structural suitability of asphalt concrete with RAP, whose environmental and economic performances support this sustainable solution.

## 2. Materials and Methods

The tested HMA, AC/31.5/sub-ballast/50–70 according to EN 13108-1 [58] identification, were composed of:

- Natural aggregate;
- RAP 0/8 mm, 8/20 mm;
- Filler;
- Bitumen with penetration grade 50/70. The bitumen content by mass of aggregate was set to 3.5–5% according to Rete Ferroviaria Italiana (RFI) specifications [59];
- The rejuvenating agent was added to the hot bitumen with a dosage of 2.3 g/kg (by weight of RAP).

**Figure 1:**
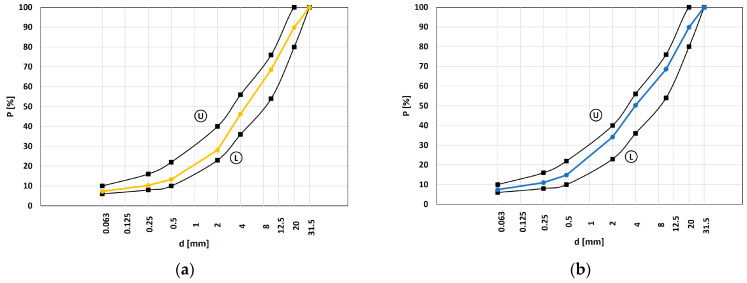

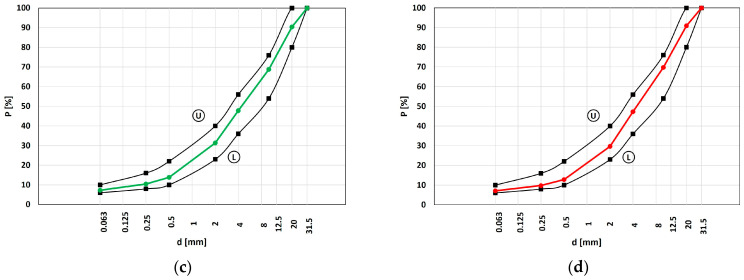
Grading curve of HMAs. (**a**) MA; (**b**) MB; (**c**) MC; (**d**) MD.

The aggregate grading (Figure 1) was designed to address the grading envelope for sub-ballast mixtures [59]. Four mixtures have been investigated (i.e., A, B, C, and D) with different RAP content (0%, 10%, 20%, and 30%, respectively) as listed in Table 1. Their grading curves are within the upper (U in Figure 1) and lower (L in Figure 1) limits.

We used RAP in two grading fractions (i.e., 0/8 and 8/20) because most of the plants are used to storing in this way. Mix B has only 10% RAP and, although we wanted to cover as much particle size as possible, it would have been impractical to use small amounts of each fraction, so we selected 0/8 RAP.

Two compaction approaches have been compared: Marshall impact compaction [60] and gyratory compaction [61].

To investigate the physical properties of the mixtures, the theoretical maximum density of the bituminous mixtures ($\rho_{mc}$) has been calculated according to [62]. The bulk density of compacted specimens (*ρ_bssd_*) has been evaluated according to the saturated surface dry method [63].

The air voids content (*Va*) has been evaluated according to [64].

Both Marshall impact compaction [60] and gyratory compaction [61] were performed to determine the OBC, comparing compaction and volumetric properties, Marshall stability, and ITS [65]. Five specimens were molded for each mixture and bitumen content (i.e., 3.5%, 4%, 4.5%, and 5%). In the manuscript, each mixture is identified using an alphanumerical code, the so-called Mijk, where i refers to the ID aggregate (I = A, ..., D), j refers to the bitumen content by mass of aggregate (j = 3.5%, 4.0%, 4.5%, and 5.0%), and k refers to the compaction process (k = M for Marshall compaction or G for gyratory compaction).

Dynamic performance tests were conducted because aged bitumen can cause higher stiffness but fragility in mixtures. Stiffness modulus and fatigue resistance were carried out to evaluate the elastic properties and durability of the mixtures. Therefore, the resistance characteristics were evaluated by carrying out dynamic stiffness tests according to [66]. Indeed, the stiffness modulus ($S_{m}$) is measured according to Equation (1):

(1) $$S_{m}=\frac{F\times \left(\nu +0.27\right)}{\left(z\times h\right)}$$

where F is the peak value of the applied vertical load; ν is the Poisson’s ratio; z is the amplitude of the horizontal deformation obtained during each load cycle; and h is the mean thickness of the specimen.

The stiffness modulus should be adjusted ($S_{m}^{\prime }$) considering a load area factor equal to 0.60 (Equation (2)):

(2) $$S_{m}^{\prime }=S_{m}\times \left(1-0.322\times \left(\log\left(S_{m}\right)-1.82\right)\times \left(0.60-k\right)\right)$$

where k is the measured load area factor.

The durability tests were performed in terms of resistance to fatigue according to [67]. In particular, the fatigue failure criterion adopted in this study is the number of repetitions needed to halve $S_{m}^{\prime }$ [68].

Finally, the economic and environmental impacts of the best-performing mixtures were investigated with a comparative life cycle analysis (LCA) with regard to the declared unit 1 Mg of the bituminous mixture. The unit price estimating took into account direct and indirect costs based on the current Italian unit prices [69]. On the other hand, the product category rules for construction products defined by [70] were implemented in SimaPro 9.3.0.3 [71] to carry out a “from cradle-to-gate” assessment [72]. The system boundary includes raw material and fuel extraction, secondary raw materials production, transportation, and mixture production in the plant [73]. The life cycle inventory analysis allowed to quantify all inputs to and outputs from the processes within the system boundaries involving several Italian producers that are representative of geographical position, use of fuels and raw materials, plant productivity, and energy consumption. The inputs for the LCA are energy and raw materials, and the outputs are emissions to air, water, and soil; solid waste generation; products; and coproducts.

Therefore, we processed inventory data about materials, transportation, and production to assess the from cradle-to-gate burdens and focused the analysis on the consumption and emission of asphalt production. Indeed, four impact categories (i.e., climate change, water use, resource use-fossils, and resource use-minerals and metals) were investigated.

## 3. Results and Discussion

### 3.1. Grading Characterization

RAP is an aged asphalt concrete crushed and selected; thus, it can be considered as unbound aggregate. Figure 1a–d shows the grading curves of MA to MD, respectively. The black lines and points define the grading envelope addressed by [59]. The RAP addition avoided basalt use in MB to MD and required decreasing the calcareous sand content from MB to MD to satisfy the grading envelope (the calcareous sand content is 30% in MA, 35% in MB, 30% in MC, and 24% in MD).

### 3.2. Volumetric Characteristics

#### 3.2.1. Theoretical Maximum Density of the Bituminous Mixtures

**Figure 2:**
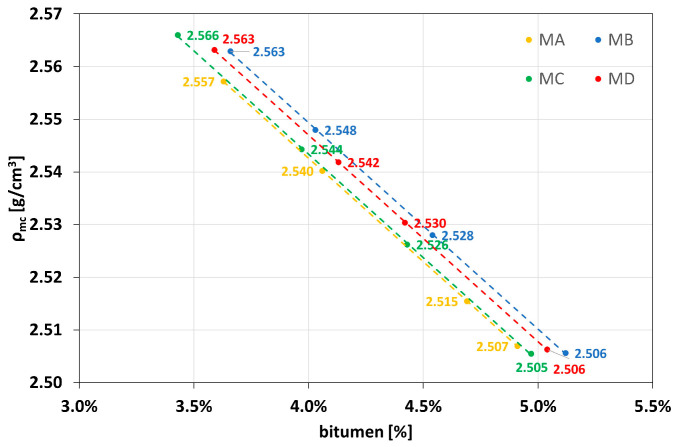
Theoretical maximum density.

Figure 2 shows the results in terms of the theoretical maximum density of the bituminous mixtures for *ρ_b_* equal to 1.02 g/cm^3^ and *ρ_a_* equal to 2.71 g/cm^3^, where *ρ_a_* is the apparent density of the aggregate, and *ρ_b_* is the density of the binder.

#### 3.2.2. Compaction and Air Voids

A total of 16 cylindrical samples were compacted according to [60] and 16 cylindrical samples were compacted with 100 gyrations according to [61].

**Figure 3:**
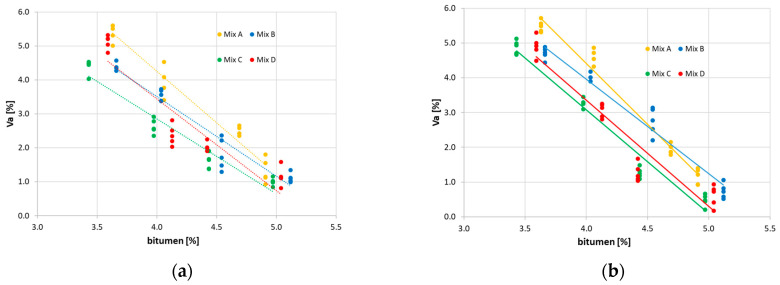
Air voids content. (**a**) MijM; (**b**) MijG.

For a given compaction method, the comparison of the results in Figure 3 highlights that the increase in RAP content implies a reduction in air voids; this is probably due to the effectiveness of the rejuvenator causing more workability.

**Figure 4:**
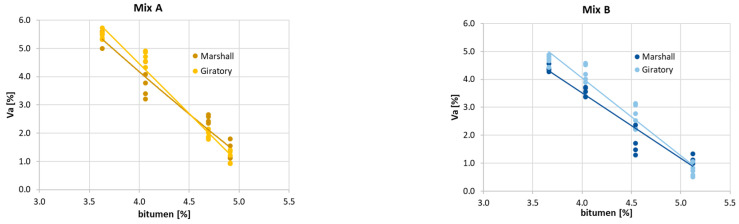

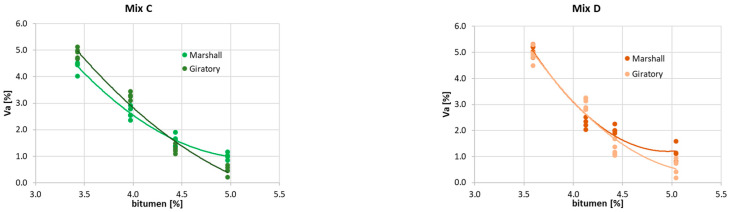
Air voids by the compaction process.

On the other hand, for each mixture, the influence of the compaction process on the mean value of air voids is not significant, especially nearby OBC (Figure 4).

### 3.3. Physical-Mechanical Properties

Marshall and gyratory specimens were tested to investigate Marshall stability and ITS.

#### 3.3.1. Marshall Stability

**Figure 5:**
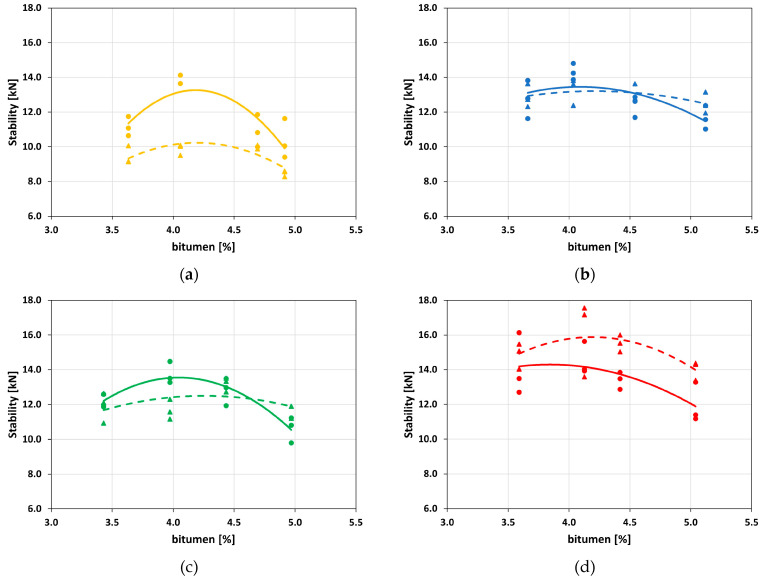
Mijk stability. (**a**) MAjk; (**b**) MBjk; (**c**) MCjk; (**d**) MDjk.

Whatever the adopted compaction process, the Marshall stability curves are smooth and convex, but the increase of RAP reverses each other’s positions. For MAjM specimens, the stability curve is higher than that of MAjG (Figure 5a); the opposite occurs for MDj (Figure 5d), while MBj and MCj (Figure 5b,d, respectively) have a breakeven point at 4.5% bitumen content.

The stability value increases with the RAP content: the presence of aged bitumen in the asphalt justifies this trend. On the other hand, the RAP content does not affect OBC which is 4.25% for all the mixtures, except for MCjG.

#### 3.3.2. Indirect Tensile Strength

**Figure 6:**
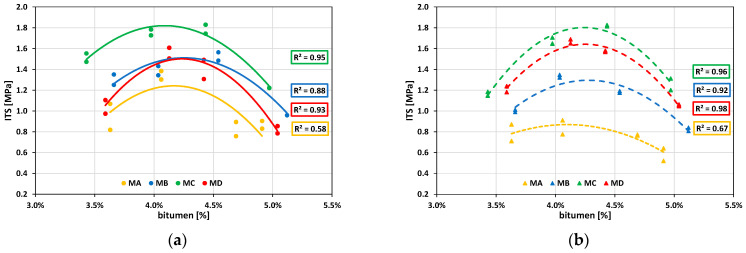
Mijk ITS curves. (**a**) MijM; (**b**) MijG.

Figure 6a,b shows the ITS curves for specimens compacted according to [60] and [61], respectively.

Figure 6 highlights that the MAjk envelope curve is the worst in terms of ITS. However, the aged bitumen from RAP may suggest excessive stiffness in MDjk, which causes mixture fragility. Indeed, according to [74], ITS increases with the RAP content due to the aged bitumen.

**Figure 7:**
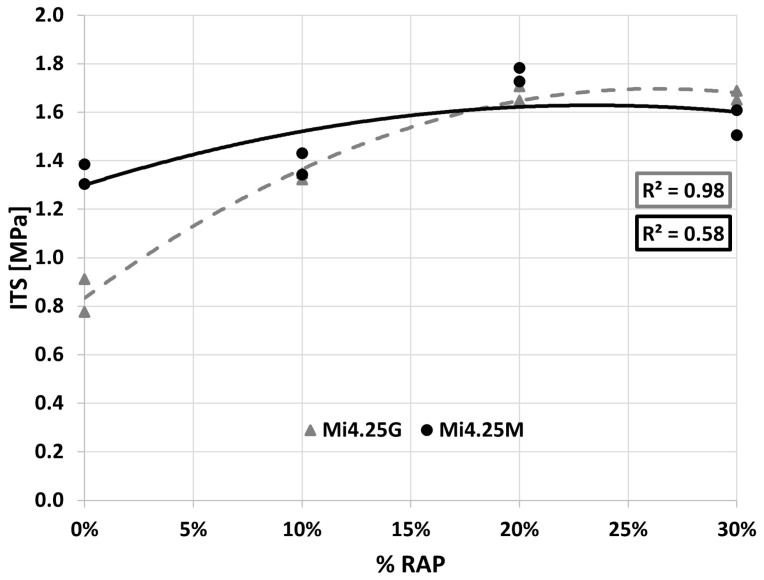
Mi4.25k ITS curves.

As regards ITS values for Mi4k, Figure 7 shows how the compaction process affects ITS when varying the RAP content for OBC. The observed trend complies with [37], where ITS increases when using up to 50% RAP.

Figure 3, Figure 4, Figure 5, Figure 6 and Figure 7 demonstrate that the investigated compaction processes give similar physical and mechanical properties in terms of *ρ_bssd_*, *Va*, Marshall stability, and ITS. OBC based on Marshall stability is 4.25% for all the investigated mixtures. Therefore, this value has been adopted in the following performance characterization carried out with specimens compacted using the gyratory compactor.

#### 3.3.3. Stiffness Modulus

Table 2 lists the stiffness modulus values (S’m) obtained at 5 °C and 25 °C for specimens at the OBC and ν equal to 0.35. For each temperature and mixture, five specimens were tested and the average results of stiffness modulus were considered.

Whatever the temperature, S’m of MD is higher than that of MA (i.e., +445 MPa at 5 °C, +226 MPa at 25 °C). The results reveal that S’m increases with the RAP content due to the higher elastic response. Nevertheless, high values of S’m could result in the fragile behavior of mixtures and low fatigue resistance [75]. On the other hand, the RAP content does not affect the reduction of S’m due to the temperature increase. Indeed, the percentage reduction is 34% for MA, 36% for MB, 38% for MC, and 36% for MD.

#### 3.3.4. Fatigue Resistance

**Figure 8:**
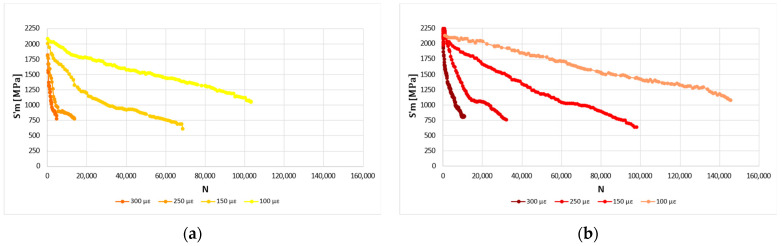
Stiffness curves’ varying ε_0_. (**a**) MA; (**b**) MD.

Fatigue tests were performed at OBC with four initial strain values (i.e., ε_0_ equal to 100 με, 150 με, 250 με, and 300 με) and were carried out on Mi conditioned at 20 °C. Figure 8a,b shows the stiffness curves’ varying ε_0_ for MA and MD, respectively.

**Figure 9:**
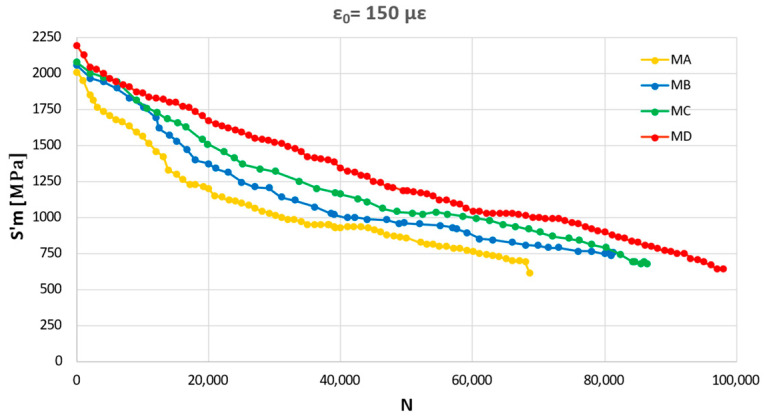
Mi stiffness curves at ε_0_ = 150 με.

Figure 9 compares the stiffness curves of MA to MD at ε_0_ = 150 με. The number of repetitions that cause fatigue failure due to half initial stiffness is 31,786 for MA, 38,569 for MB, 48,499 for MC, and 57,061 for MD.

A more in-depth analysis reveals:

- ε_0_ affects the slope of the stiffness curves and then the fatigue resistance because the more the ε_0_ value, the more the slope (Figure 8);
- MD shows higher fatigue resistance than the other mixtures (Figure 9). Whatever Mi, the mixtures with higher RAP content show significant fatigue resistance for all values of ε_0_ (Table 3). Indeed, N for MA at ε_0_ equal to 100 με is 103,259, while N for MD at ε_0_ equal to 100 με is 145,677. The trend of MB and MC is consistent with the results of MA and MD. The causes could be linked to the effectiveness of the additive on old bitumen and on the compaction processes;
- For a given ε_0_, S’m increases with the RAP percentage (i.e., for ε_0_ equal to 250 με S’m is 1806 MPa for MA, 1947 MPa for MB, 2047 MPa for MC, and 2163 MPa for MD). From MA to MD, the percentage increase is 7.2% for ε_0_ equal to 300 με, 19.8% for ε_0_ equal to 250 με, 9.1% for ε_0_ equal to 150 με, 1.9% for ε_0_ equal to 100 με.

**Figure 10:**
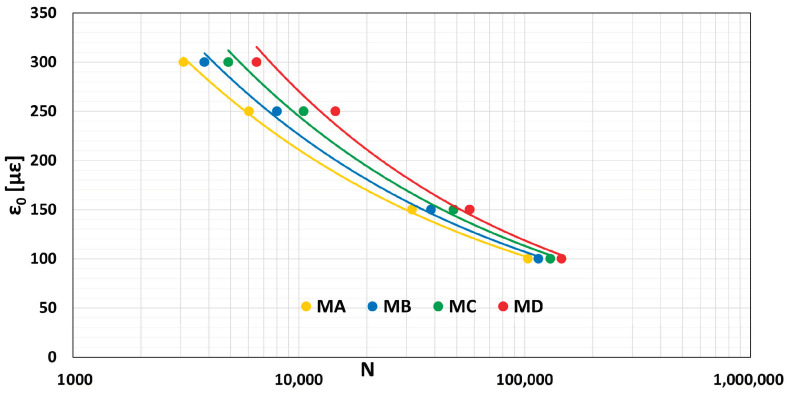
Mi4.25G fatigue curves.

Figure 10 represents the fatigue curve of each tested mixture.

When modifying the mixtures in terms of RAP and of rejuvenator additive, an improvement in the durability of the compounds and a consequent increase in fatigue strength are observed. Specifically, Equation (3) describes the relationship between the number of load applications and the tensile strain.

(3) $$N=k_{1}\times \varepsilon_{0}{}^{k2}$$

where $k_{1}$ and $k_{2}$ are the regression coefficients identified for each Mi, and ε_0_ is the tensile strain in με at the center of the specimen. Then, the regression Equations (4) to (7) for MA to MD are, respectively:

(4) $$N=1.882\times 10^{-8}\times \varepsilon_{0}{}^{-3.190}$$

(5) $$N=1.143\times 10^{-8}\times \varepsilon_{0}{}^{-3.279}$$

(6) $$N=1.958\times 10^{-8}\times \varepsilon_{0}{}^{-3.244}$$

(7) $$N=1.484\times 10^{-7}\times \varepsilon_{0}{}^{-3.034}$$

where the coefficients of determination are equal to 0.999, 0.996, 0.994, and 0.986 for MA to MD, respectively.

### 3.4. Analysis of the Production Costs

Given the experimental results, the production costs of the tested mixtures at OBC were assessed with regard for the current Italian prices of:

1. Natural raw materials (i.e., aggregates and bitumen), secondary raw materials (i.e., RAP), and products (i.e., rejuvenating agent);
2. Energy (i.e., fossil fuels and electricity);
3. Overhead, contingencies, miscellaneous, profit, and value-added tax (i.e., indirect costs).

The unit price estimating refers to the reference unit 1 Mg. Table 4 lists the results.

The cost production of MA is the highest one (i.e., 49.96 €/Mg) because it has the maximum content of virgin bitumen that accounts for 45% of the MA total cost and 34% of that of MD. Therefore, the overall unit price of Mi decreases with the RAP content compared to MA (−5.4% for MB, −9.4% for MC, and −14.1% for MD). On the other hand, the RAP addition does not affect the mixing temperature and fuel consumption.

### 3.5. Assessment of the Environmental Impacts

**Figure 11:**
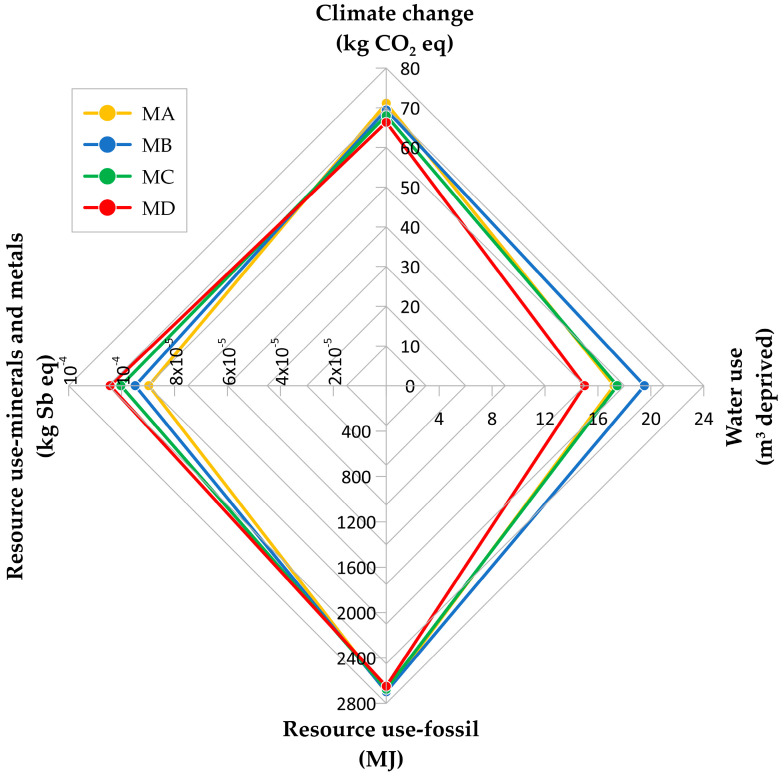
Mi LCIA results.

The LCIA allowed a comparison of the environmental performances of the tested mixtures at OBC. Figure 11 shows the results in terms of climate change (kg CO_2_ eq), water use (m^3^ depriv.), resource use-fossils (MJ), and resource use-minerals and metals (kg Sb eq) [76].

The LCIA results suggest that the use of RAP can bring benefits in terms of energy savings, environmental impact, and minimization of the use of resources. With regard to climate change, the production of MA implies 71.14 kg CO_2_ eq, while MB is 69.45 kg CO_2_ eq, MC is 68.03 kg CO_2_ eq, and MD is 66.32 kg CO_2_ eq. It leads to an emissions reduction of greenhouse gases equal to up to 4.82 kg CO_2_ eq/Mg (−6.8%). Additionally, considering water and resource fossils use, MD has lower impacts than MA (14.99 m^3^ depriv./Mg and 17.22 m^3^ depriv./Mg, and 2649 MJ/Mg and 2704 MJ/Mg, respectively). The percentage differences are −12.9% for water use and −2.0% for fossils consumption. However, the calcareous sand content drives the water consumption which is 19.52 m^3^ depriv./Mg for MB and 17.46 m^3^ depriv./Mg for MC. On the other hand, resource use-minerals and metals shows a counter trend compared to other impact categories because it increases with the RAP and rejuvenator content (i.e., 8.97 × 10^−5^ for MA, 9.49 × 10^−5^ for MB, 1.00 × 10^−4^ for MC, and 1.04 × 10^−4^ for MD). In particular, the rejuvenator accounts for 13%, 26%, and 38% of resource use-minerals and metals for MB to MD, respectively.

## 4. Conclusions

The Italian railway company uses sub-ballast under the ballasted track to reduce stress–strain conditions on the unbound layers and prevent contamination between ballast and subgrade. This study assessed the mechanical, environmental, and economic performances of asphalt mixtures containing 0% to 30% RAP. The addition of reclaimed asphalt pavement forces the use of a rejuvenating agent due to the aged bitumen characteristics.

The current Italian specifications for railway sub-ballast require the Marshall mix design and indirect tensile strength. To validate the volumetric mix design as European standards require, the bulk density, the air voids content, the Marshall stability, and the indirect tensile strength were tested with specimens compacted according to [60] and compared with the results from specimens molded with a gyratory compactor according to [61]. The results proved the compaction process does not significantly affect OBC that is 4.25%; then, the gyratory compaction method can be successfully used for mix-design. The main conclusions were drawn as follows:

- Physical and mechanical tests revealed how a higher RAP content increases Marshall stability (9.5 kN with 0% RAP vs. 16.6 kN with 30% RAP) and indirect tensile strength (0.91 MPa with 0% RAP vs. 1.65 MPa with 30% RAP).
- S’m increases with the RAP content (e.g., at 25 °C MA has S’m equal to 1744 MPa and MD has S’m equal to 1970 MPa).
- The fatigue tests with four initial strain values (i.e., 100 με, 150 με, 250 με, and 300 με) confirmed that the increase in stiffness modulus corresponds to an increase in terms of durability, and the tested mixtures are well-suited for railway sub-ballast (e.g., at initial strain equal to 150 με, N for MA is 31,786 and for MD is 57,061).
- The economic and environmental analyses of the mixtures with OBC demonstrated that those with the highest RAP content have the lowest unit price (42.92 €/Mg for MD instead of 49.96 €/Mg for MA) and the lowest burdens in terms of climate change, use of water, and resource-fossils (−6.8%, −12.9%, and −2.0%, respectively). The decreasing bitumen and natural aggregate content justifies these results, while the rejuvenating agent causes the increase in resource use-minerals and metals (+15.9%).

This research focused on laboratory characterization of asphalt concrete with RAP, and further investigation shall be carried out to monitor built sub-ballast layers exposed to railway traffic. Further analyses shall involve the structural properties of this material with regard to its fatigue resistance and dynamic performances under actual traffic, weather, and environmental conditions. Comparative structural trackbed analyses with elastic multilayer software shall investigate rail track degradation due to fatigue and rutting to schedule maintenance program activities and predict their life cycle costs.

## Figures and Tables

**Table 1:** Aggregates composition.

ID Aggregate	Natural Aggregates [%]	RAP 0/8 [%]	RAP 8/20 [%]
A	100	0	0
B	90	10	0
C	80	10	10
D	70	15	15

**Table 2:** Average stiffness moduli value.

Mi	S’m [MPa] 5 °C	S’m [MPa] 25 °C
MA	2651	1744
MB	2740	1751
MC	2985	1857
MD	3096	1970

**Table 3:** Results of the fatigue tests.

Mi	ε_0_ [με]	S’m (ε_0_) [MPa]	N (-)
MA	100	2084	103,259
	150	2006	31,786
	250	1806	6021
	300	1819	3090
MB	100	2079	115,013
	150	2053	38,569
	250	1947	8007
	300	1667	3824
MC	100	2091	130,071
	150	2075	48,499
	250	2047	10,507
	300	1948	4879
MD	100	2124	145,677
	150	2188	57,061
	250	2163	14,527
	300	1950	6508

**Table 4:** Unit price estimating.

Mi	Raw Materials €/Mg	Energy €/Mg	Indirect Costs €/Mg	Total €/Mg
MA	22.68	13.18	14.10	49.96
MB	19.97	13.18	14.10	47.25
MC	17.96	13.18	14.10	45.24
MD	15.64	13.18	14.10	42.92

## Data Availability

The data presented in this study are available on request from the corresponding author. The data are not publicly available due to confidentiality reasons.
